# Reoviral Hepatitis in Young Turkey Poults—An Emerging Problem

**DOI:** 10.3390/pathogens14090865

**Published:** 2025-09-01

**Authors:** Rahul Kumar, Mohamed Selim, Anibal G. Armien, Sagar M. Goyal, Fabio A. Vannucci, Sidhartha Deshmukh, Robert E. Porter, Sunil K. Mor

**Affiliations:** 1 Minnesota Veterinary Diagnostic Laboratory, Veterinary Population Medicine Department, College of Veterinary Medicine, University of Minnesota, Saint Paul, MN 55108, USA; kumar509.umn@gmail.com (R.K.); agarmien@ucdavis.edu (A.G.A.); goyal001@umn.edu (S.M.G.); vannu008@umn.edu (F.A.V.); sdeshmuk@umn.edu (S.D.); porte349@umn.edu (R.E.P.); 2 Tennessee Department of Agriculture, KAHDL, Nashville, TN 37220, USA; 3 Animal Disease Research & Diagnostic Laboratory, Veterinary and Biomedical Sciences Department, South Dakota State University, Brookings, SD 57007, USA; mohamed.selim@sdstate.edu; 4 California Animal Health and Food Safety Laboratory System, Davis Branch, School of Veterinary Medicine, University of California, Davis, CA 95616, USA

**Keywords:** turkey hepatitis reovirus, poult, mortality, liver, lameness, turkey reoviruses, hepatitis

## Abstract

From January 2019 to April 2020, the Minnesota Veterinary Diagnostic Laboratory (MVDL) received cases of hepatitis and spiking mortality in young turkey poults (average age 15.5 days) from multiple turkey-producing states. Meat-type turkeys were mainly affected, with a few cases in breeders. Of 188 cases, 88 (47.5%) tested positive for reovirus by virus isolation, with most of the positive cases from 7 to 14-day-old birds (n = 42). Gross lesions consisted of hepatosplenomegaly with acute, multifocal necrosis in both liver and spleen. Microscopically, liver sections showed congestion of hepatic sinusoids and necrotizing hepatitis with infiltration of lymphocytes, plasma cells, and macrophages. Reovirus was detected in liver samples by electron microscopy and in situ hybridization (ISH). Sections of spleen showed areas of necrosis with infiltration of the mixed population of inflammatory cells and depletion of lymphocytes. We consistently isolated reoviruses from livers and tentatively named the virus “Turkey Hepatitis Reovirus” (THRV). Phylogenetic analysis of the newly emerged THRVs revealed their clustering into four distinct groups. This study also highlighted the close antigenic relation between TARV and THRV compared to turkey enteritis reoviruses (TERVs), which shed light on the probable origin of this newly emerged pathotype. In summary, further molecular and pathogenicity studies are recommended on THRVs to help diagnose and control this serious variant.

## 1. Introduction

Avian reovirus belongs to the genus Orthoreovirus within the family Reoviridae and infects several avian species (https://ictv.global/taxonomy; accessed on 21 March 2024). It is a 70–80 nm, non-enveloped, icosahedral virus with a segmented, double-stranded RNA genome [1]. Avian reoviruses (ARVs) are ubiquitous in domestic poultry, with 80% of them being non-pathogenic [2]. The avian reovirus genome consists of 10 segments, which are classified according to their size into large (L1, L2, and L3), medium (M1, M2, and M3), and small (S1, S2, S3, and S4) segments. All segments are monocistronic except for S1, which is tricistronic. It encodes three viral proteins: two nonstructural proteins (p10 and p17), and one structural protein, Sigma C (σC) [3]. The σC protein is a minor outer-capsid structural protein translated by the third-largest open reading frame (ORF) of the S1 segment [4]. It is composed of 326 amino acids, which are divided into two variable regions: the N-terminal region (1 to 122 amino acids) and the C-terminal region (196 to 326 amino acids). It forms a homotrimer responsible for cell attachment [5] and antigenicity, as it is the main site of the type- and broad-specific neutralizing epitopes [6]. Sigma C is commonly used for genotypic classification and characterization of ARVs [7,8].

Clinical disease associated with ARVs mostly depends on the virus pathotype, route of exposure, age, and immune status of the affected bird [2]; younger birds are more susceptible to infection with this virus [9]. ARV infects most avian species and has been associated with a wide range of diseases, e.g., tenosynovitis, respiratory disease, enteric disease, hydropericardium, hepatitis, splenitis, and runting/malabsorption syndrome in chicken [2,10], duck [11,12,13], goose [14,15,16], turkey [17,18,19] quail [20], and wild birds [21,22].

In turkeys, reovirus is mainly associated with enteritis and arthritis. The viruses associated with these disease conditions are tentatively known as turkey enteric reoviruses (TERVs) and turkey arthritis reoviruses (TARVs), respectively. The TERVs have been isolated for years from the feces/intestines of apparently healthy poults and cases of poult enteritis [19,23,24]. In the 1980s, reovirus was detected in cases of arthritis in turkeys [25,26,27]. No further reports of reovirus-associated arthritis in turkeys were available until the disease reappeared in 2011 [28]. Currently, TARV is one of the top ten diseases, which has a detrimental impact on one of the most promising industries in the US, with highly divergent strains circulating in the country [29]. After the emergence of TRVs, the incidence of reported cases increased significantly. Despite the absence of commercial vaccines specific to TRV, autogenous vaccines, prepared from the circulating strains, were used in breeder flocks. This approach helped prevent infection in breeders and transferred passive immunoglobulins to the newly hatched poults. Recently, a third type of turkey reovirus, which causes hepatitis and mortality in poults, emerged in 2019 [30,31,32]. This disease is characterized by spiking mortality, hepatitis, and occasional splenitis. An experimental study from our lab, using reovirus isolates from cases of hepatitis and splenitis, orally inoculated one-week-old turkey poults from a reovirus infection- and vaccination-free breeder flock, established Koch’s postulates for this disease [33]. This virus has tentatively been named “Turkey Hepatitis Reovirus” (THRV). The present study reports the isolation of THRV, evaluation of trends in geographical distribution, seasonality, age susceptibility, and diagnostic testing. Additionally, this study represents the first molecular characterization of these novel emergent variants of avian reoviruses.

## 2. Materials and Methods

### 2.1. Case Definition

Reovirus was considered the confirmed cause of death if the dead bird had hepatosplenomegaly with histopathologic evidence of hepatic and/or splenic necrosis with the detection of reovirus by one or more of the three tests, e.g., real-time reverse transcription-PCR (rRT-PCR) [33], virus isolation (VI) in cell cultures and/or specific-pathogen-free (SPF) embryonated chicken eggs, and in situ hybridization (ISH) on liver sections.

### 2.2. Retrospective Data Review

The electronic database of the Minnesota Veterinary Diagnostic Laboratory (MVDL) was queried, and cases of turkeys with liver and/or spleen lesions submitted from January 2019 to April 2020 were selected. The information included details about several different clients, their geographical locations, the age of the birds, clinical histories, and laboratory test results. The comprehensive diagnostic work on 185 cases included necropsy, gross and microscopic examinations, virology, and bacteriology. In some cases, electron microscopy and ISH were also performed.

### 2.3. Pathology

At necropsy, the liver, spleen, lungs, intestine, and other visceral organs showing gross lesions were collected for routine histopathology from cases mentioned in the electronic database from January 2019 to April 2020. All tissues were fixed in 10% neutral buffered formalin, trimmed, embedded in paraffin, sectioned at 4 μm, mounted on glass slides, and stained with hematoxylin and eosin (H&E). Sections of livers from some cases were tested for the presence of viral mRNA by ISH as described below.

### 2.4. In Situ Hybridization

The ISH was performed using a specific probe for the *S2* gene, utilizing the RNAscope 2.5 HD detection red kit (Advanced Cell Diagnostics, Newark, CA, USA) [34]. Liver samples negative for reovirus by rRT-PCR and virus isolation were used as negative controls. Briefly, FFPE sections were typically deparaffinized by immersing them in xylene for 2–3 cycles, with each immersion lasting between 5 and 10 min. Endogenous peroxidase was inactivated by 3% hydrogen peroxide for 10 min at room temperature. The sections were then boiled for 15 min in RNAscope target retrieval buffer, followed by 15 min of incubation at 40 °C with RNAscope protease reagent. Hybridization with the *S2* gene probe was performed at 40 °C for 2 h. Slides underwent a series of amplification steps as recommended by the manufacturer. The signals were detected with Fast Red. Slides were counterstained with 50% Gill’s hematoxylin I for 30 s and mounted with VectaMount. Sections were then examined under a bright-field microscope.

### 2.5. Bacterial Isolation

Swabs from liver and spleen were routinely investigated for bacteriology and *Mycoplasma* spp., as part of the standard diagnostic panel at the Minnesota Veterinary Diagnostic Laboratory (MVDL). Briefly, the swabs from liver and spleen were cultured on sheep blood agar and MacConkey agar (Becton, Dickinson, ND, USA). Swabs were tested also for the presence of Mycoplasma by incubation in Frey’s broth at 37 °C in a 5% CO_2_ atmosphere (Becton, Dickinson, ND, USA), followed by plating on agar plates and incubation at 37 °C for 2 weeks [35]. Real-time PCR was used for confirmation of Mycoplasma (https://vdl.umn.edu/laboratories/molecular-diagnostics, accessed on 21 March 2024).

### 2.6. Virus Isolation

Homogenates of liver were inoculated in SPF embryonated chicken eggs (ECE) as described previously [23]. Briefly, a 10% suspension of pooled liver samples in Hanks’ balanced salt solution (HBSS) was homogenized in a stomacher. The homogenates were centrifuged at 1500× *g* for 15 min, and the clear supernatants were mixed with 1% Penicillin–Streptomycin and 1% Fungizone, followed by inoculation in 6-day-old ECE via the yolk sac route and incubation at 37 °C. After four days of incubation, the yolk sac and embryo homogenates were processed for blind passage. A total of 1–2 blind passages were given for each sample. Additionally, supernatants were inoculated on QT-35. Inoculated cells were incubated at 37 °C and examined daily under an inverted microscope for the appearance of reovirus-specific cytopathic effects (CPEs) [24]. Up to three blind passages were given in cell cultures.

### 2.7. Transmission Electron Microscopy (EM)

The fresh and frozen tissue samples were prepared for virus screening and identification by EM as described previously [36]. Purified virus suspension from liver samples was added to a formvar-coated copper grids and contrasted with 1% phosphotungstic acid (Electron Microscopy Sciences). For tissue ultrastructure, formalin-fixed liver samples were post-fixed in 2.5% glutaraldehyde in 0.1 M sodium cacodylate buffer and 1% osmium tetroxide in the same buffer. The tissue samples were then dehydrated in a graded series of alcohols and embedded in resin. Following trimming, thin sections (60–70 nm thick) were obtained using an ultramicrotome (Leica Microsystems, Vienna, Austria). Samples were then negatively stained and examined under a 1200 EX II transmission electron microscope (JEOL Ltd., Tokyo, Japan). Images were obtained using an AMT Capture Engine Version 7.00 camera and software (Advanced Microscopy Techniques Corp, Woburn, MA, USA). Image analysis was carried out using ImageJ (NIHR public domain, https://imagej.net/ij/).

### 2.8. σC Gene Sequencing

The positive samples for universal RT-PCR and virus isolation underwent a specific PCR assay to amplify the entire length (~900 nucleotide long fragment) of the σC in the S1 segment, as previously mentioned by Gál et al. [37]. The gel bands showed the expected band size of the σC, which were extracted and subjected to Sanger sequencing using the BigDye Terminator v3.1 Cycle Sequencing Kit.

### 2.9. Phylogenetic Analysis

The THRV isolates (n = 25) from the US and Canada were sequenced for genotyping characterization using the previously described classification [7,8]. Phylogenetic analysis was conducted by comparing the σC gene of THRVs from this study, as listed in Table 1, with representative turkey and avian reovirus sequences downloaded from GenBank. Multiple sequence alignment was performed using ClustalW in Clustal Omega software [38], and the identity percentage was calculated among the various sequences. Furthermore, multiple sequence alignment with distance analysis was also performed on all sequences in this study using the Muscle algorithm within the Geneious Prime software Version 5.5. [39]. Finally, a maximum likelihood tree was generated, and its robustness was statistically validated through 1000 bootstrap replicates using the best-fit substitutional models, as implemented in MEGA11 software (version 11.0.13) [40].

## 3. Results

### 3.1. Retrospective Data Summary

The geographic distribution of submissions from the US and Canada is shown in Table 2. Of the 185 cases, 176 were from 10 states in the US and 9 were from Canada. Of the 176 cases from the US, 122 (69%) were from Minnesota. A large majority of cases (164 of 185 or 89%) were from birds less than 21 days of age, while only 21 (11%) were from older birds (Table 3).

**Figure 1:**
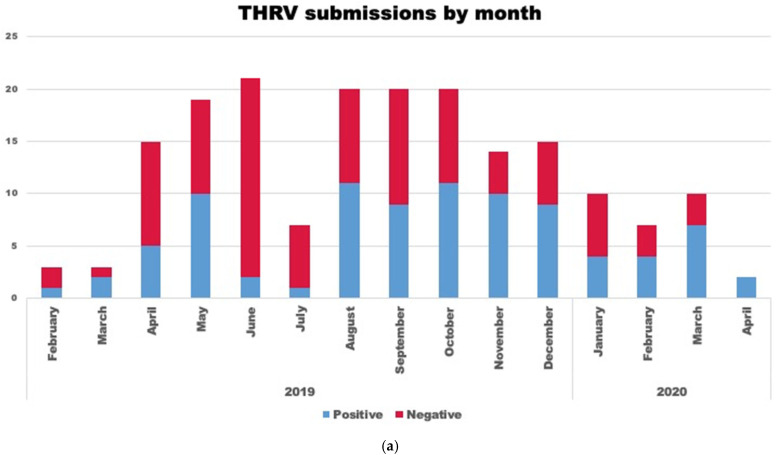

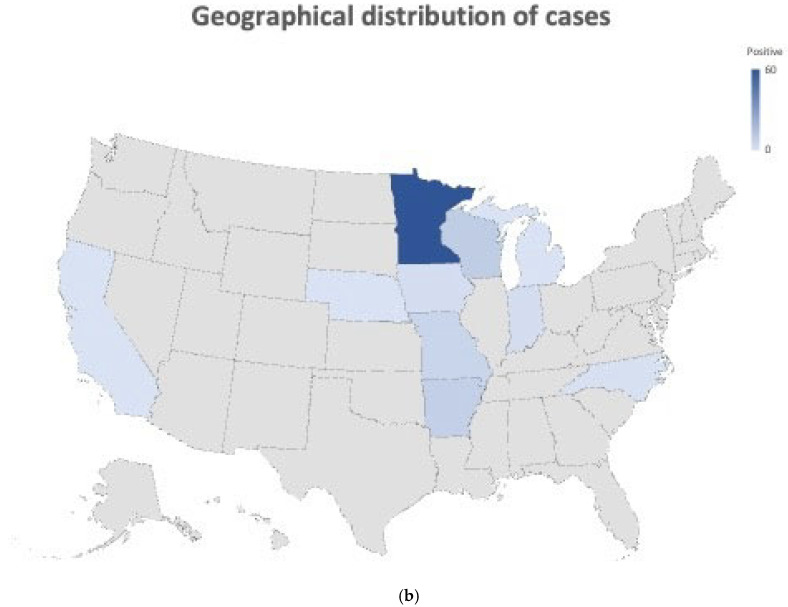
(**a**) Stacked bar graph representing a month-wise number of turkey hepatitis reovirus (THRV)-positive (blue bar) and negative (red bar) cases out of the total number (red + blue) of cases submitted. (**b**) Figure showing the geographical and state-wise distribution of THRV-positive cases received from different states in the US. A darker shade of blue means a higher number of THRV-positive cases received at MVDL.

A total of 88 (47.5%) cases tested positive for reovirus by virus isolation. Most of the virus-positive cases were from 7 to 21-day-old birds (n = 69) (78.4%) with an average age of 15.5 days. Reovirus was isolated after one (n = 74) or two passages (n = 10) in QT-35 cells. Four cases were only positive in ECE after two passages. Month-wise submission data and the geographical distribution of submissions are shown in Figure 1a and Figure 1b, respectively. There appears to be no seasonal effect of this condition, but there was an increase in total submissions and positive cases from August to December 2019 (Figure 1a).

### 3.2. Gross Lesions and Histopathology

**Figure 2:**
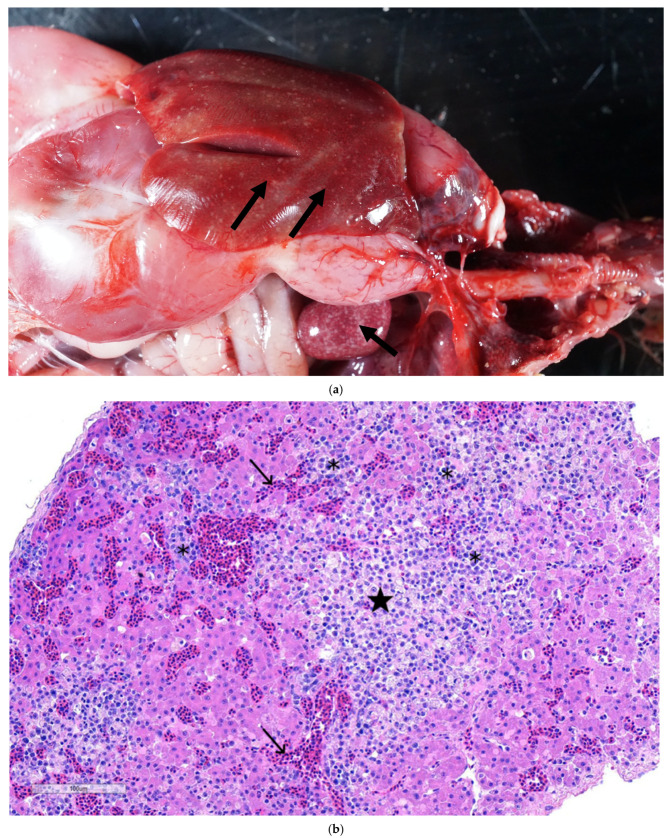

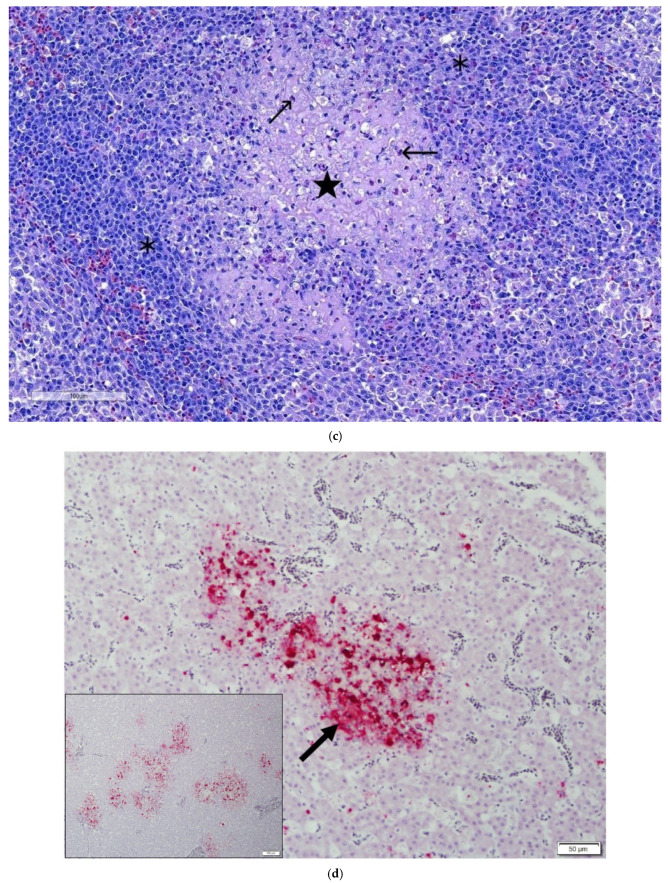
(**a**) Turkey liver and spleen: showing white necrotic foci in a turkey poult’s hepatic (arrow) and splenic parenchyma (arrow). (**b**) Section of liver showing plum islands of hepatic necrosis (★) having lymphoplasmacytic cell infiltration (✱) and congestion of hepatic sinusoids. **H&E. 10×**. (**c**) Section of spleen showing necrotizing splenitis, characterized by an area of necrosis (★) evidenced by the absence of nuclei and the presence of ghost cells (karyolysis). A mixed population of inflammatory cells (✱). Lymphoid depletion is apparent and evident by numerous apoptotic bodies (→) observed within lymphoid follicles. H&E. (**d**) Section of liver demonstrating intense red color as a positive ISH reaction showing reoviral RNA by in situ hybridization in intralesional cells (arrowheads) in liver. Inset showing TRV RNA localized to discrete foci in areas of hepatocellular necrosis with inflammation.

At necropsy, hepatosplenomegaly was evident, with widespread areas of focal necrosis in the hepatic and splenic parenchyma (Figure 2a). Microscopically, liver sections showed necrotizing hepatitis with infiltration of lymphocytes, plasma cells, and macrophages, and congestion of hepatic sinusoids (Figure 2b). Fibrin accumulation was observed in necrotic areas with occasional bile duct hyperplasia in some sections. Sections of spleen showed areas of necrosis with infiltration of a mixed population of inflammatory cells and depletion of lymphocytes (Figure 2c). There was prominent reticular cell hyperplasia and proliferation with marked erythrophagocytosis and numerous siderophages in some sections. Sections of the lungs showed interstitial pneumonia, characterized by distension of blood capillaries with erythrocytes, cell debris, and mononuclear cells.

### 3.3. In Situ Hybridization

The ISH probe, specifically designed to target reovirus, showed a positive in situ hybridization reaction (Figure 2d) surrounding areas of necrosis and cellular infiltration. The positive reaction is multifocal and diffused throughout the areas of hepatic necrosis in the liver parenchyma (inset Figure 2d). The positive ISH reaction, demonstrating the presence of reoviral mRNA in the hepatic parenchyma, suggests that reovirus was directly associated with the characteristic hepatic lesions.

### 3.4. Bacterial Isolation

There was no significant bacterial growth from the liver, spleen, intestine, or lungs, but low numbers of *Enterococcus* sp. and Klebsiella pneumoniae were isolated from the liver, lung, and trachea of one case. No *Salmonella* spp. was isolated.

### 3.5. Virus Isolation

The embryos inoculated with tissue homogenates showed stunting, hemorrhages, and death of the embryo. The allantoic fluid and stunted embryos were processed for the next passage. Most of the reovirus-positive samples caused the death of the embryo in the first two passages. Allantoic fluid and stunted/dead embryos were tested for reovirus by rRT-PCR. Some of these fluids were also subjected to negative contrast electron microscopy. In reovirus-positive cases, characteristic syncytia were seen in QT-35. No adenovirus or picornavirus was isolated from liver samples.

### 3.6. Transmission Electron Microscopy

**Figure 3:**
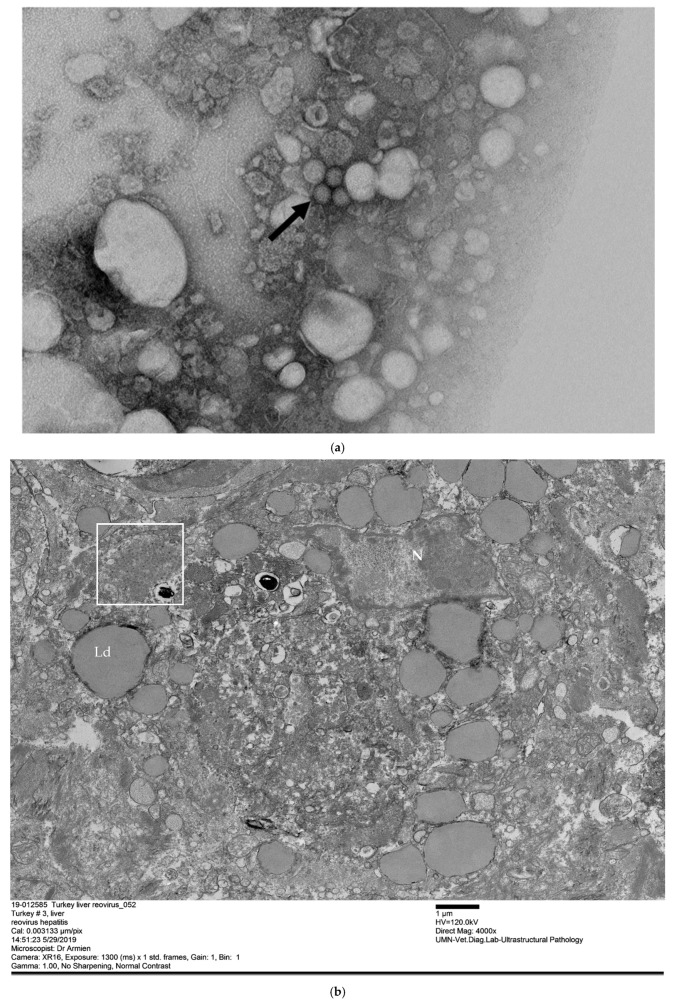
(**a**) Non-enveloped, spherical ~70–80 nm reovirus particles (arrowhead) extracted and concentrated from liver tissue. Transmission electron microscopy, scale bar 200 nm. (**b**) Liver, hepatocyte with cytoplasmic replication, and assembly organelle with reovirus virion (box). The hepatocyte contained lipid droplets (Ld) and is surrounded by fibrin. Transmission electron microscopy.

Ultrastructurally, non-enveloped, ~70 to 80 nm virions with icosahedral symmetry, consistent with members of the family Reoviridae, were detected on a negatively contrasted preparation (Figure 3a). Formalin-fixed liver samples revealed acute swelling and necrosis of hepatocytes with disruption of the vascular bed and fibrin deposits surrounding the necrotic hepatocytes. Numerous virus particles were observed in the necrotic debris and within the necrotizing hepatocytes (Figure 3a). The virions were distributed in a moderate electron-dense material and arranged in small groups (Figure 3b).

### 3.7. Phylogenetic Analysis

**Figure 4:**
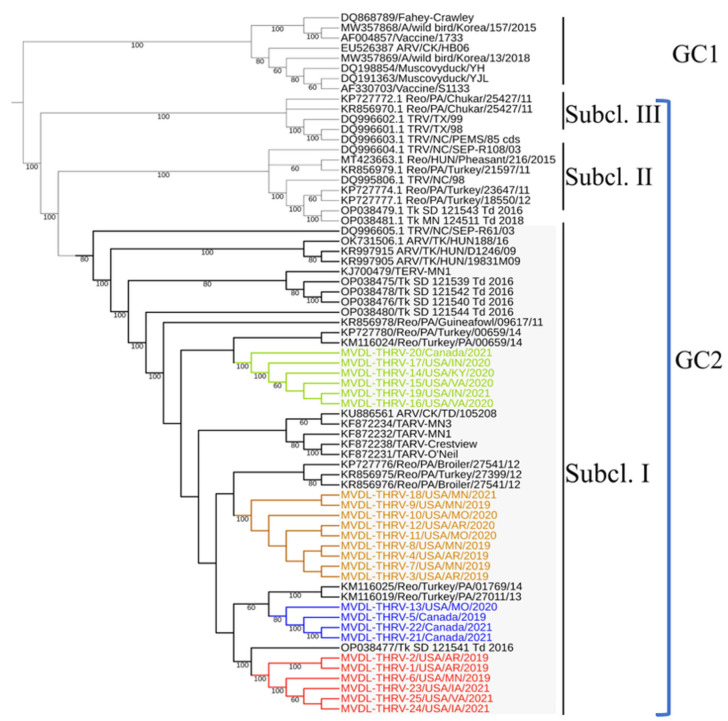
A maximum likelihood (ML) tree was generated based on the nucleotide sequences of the complete σC gene of 25 THRV sequences and representative turkey and avian reovirus sequences from GenBank. Tamura-Nei (TN93 + G) was the best-fitting nucleotide substitution model used for reconstructing the ML tree and was validated by 1000 bootstrap replicates. Genetic cluster 1 (GC1) and genetic cluster 2 (GC2). Subcluster I (Subcl. I), subcluster II (Subcl. II), and subcluster III (Subcl. III). Four distinct groups of THRVs were highlighted with different colors. Group I is marked in red, Group II in blue, Group III in yellow, and Group IV in green.

The phylogenetic analysis based on the complete σC gene was performed using the maximum likelihood method, with high bootstrap values supporting the results. All sequences (THRVs and reference sequences) used in this study were divided into two main genetic clusters (GCs) (Figure 4). GC1 contained the classical reoviruses, including wild birds and ducks, and attenuated vaccines (S1133 and 1733), while GC2 included all TRVs (TERV, TARVs, and THRVs) and some CARVs. GC2 was further divided into three subclusters: I, II, and III. The 25 THRV sequences were grouped into subcluster I and further subdivided into four groups based on 3% or more nucleotide (nt) and amino acid (aa) identity differences. The intra-group average identity percentages were more than 99%. Inter-group average identity percentages were around 96 to 97% nucleotide (na) and amino acids (aa) (Table 4). Group I included six strains from, MN, AR, and VA. Group II had four strains (three from Canada and one from MO). Nine sequences from MN, AR, and MO were clustered in group III, and group IV included six strains from Canada, KY, VA, and IN. In addition to THRVs, subcluster I had different chicken and turkey arthritis and enteritis reovirus sequences. Some of these reference strains were identical to the THRVs, while the others showed relative divergence. Interestingly, the TARV sequence from SD (Tk_SD_121541_Td_2016) shared 98.7% nt and 99.0% aa identity with group 1 THRVs, making it the only closely related sequence reported so far in the GenBank. The two TARVs from PA (Reo/Turkey/PA/01769/14 and Reo/Turkey/PA/27011/13) shared the same node with group II THRVs, with 98.3 nt and 98.4% aa identities (Table 4). Three out of the total four THRVs from Canada were clustered in group II. Similarly, turkey and chicken reovirus strains from Pennsylvania (Reo/PA/Turkey/27399/12 and Reo/PA/Broiler/27541/12) were also included in the same nodes of groups III and IV, with ~98.0% identity (Table 4). The reference TARV O’Neil and related TARVs were also closely related to THRVs, mainly with groups 11–1 V with ~98.0% identities (Figure 4). The identity percentages between the THRV groups and the subclusters II and III avian and turkey reoviruses were around 90–91% and 86–87%, respectively, dropping sharply to 59% na and 54% aa with the ARVs in GC 2 (Table 4).

**Figure 5:**
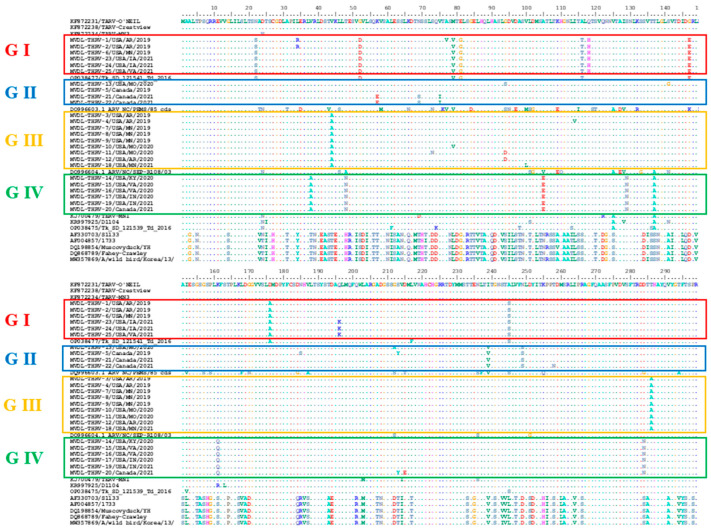
Deduced amino acids alignment of the σC protein of 25 THRVs and representative sequences of turkey and avian reoviruses. Specific color frames are representative of different groups of THRVs.

The deduced aa alignment of σC protein between the THRVs and the representative TRVs and ARVs also confirmed the maximum likelihood tree topology and average identity percentage (Figure 5). The unique aa substitutions were detected in each THRV group. The group I THRVs possessed nine aa substitutions (22S, 52N, 79V, 81G, 116T, 118H, 147E, 176A, 245S), and group II had two aa (239V and 249S) in all sequences, but two sequences had three extra substitutions 57E, 69S, and 75I. The characteristic aa substitutions in group III were 44A, and 286A while group IV had seven aa substitutions (38A, 48N, 105E, 129N, 137A, 161Q, and 284N). These unique substitutions were exclusive for THRV groups and were absent in all other TRVs and ARVs, except those sharing the same nodes with the groups that had one or two amino acids in common with these groups. Exceptionally, the Tk_SD_121541_Td_2016 strain, which is highly identical to group I, has all the characteristic amino acids of group I except Q118H, plus it also has L217F. The THRV groups exhibited several nonsynonymous mutations compared to other AVRs and TRVs within subcluster I, with the highest number of mutations observed compared to TRV/NC/SEPR61/03. Interestingly, the TERV-MN1 has unique aa mutations D24N, N69D, Q122R, V125A, L230M, V214I, and N236S, hence differentiating it from TARVs and THRVs.

## 4. Discussion

The results of the present study, together with those of an earlier experimental study fulfilling Koch’s postulates [33], establish that turkey reovirus can cause not only enteritis and arthritis but also hepatitis and splenitis, leading to spiking mortality in young turkey poults. Since the early 1990s, turkey reovirus seems to have been evolving in terms of its pathogenicity [25,26]. TERV was a cause of concern for years [19,23,24,41]. Subsequently, lameness caused by TARV re-emerged a decade ago, resulting in substantial economic losses due to non-uniformity of the flock, mortality, and condemnation of carcasses in the grow-out phase [28]. This study reports another condition associated with turkey reovirus, i.e., hepatitis in <3-week-old poults, characterized by spiking mortality and necrotizing hepatitis and splenitis. Recently, considering the notable rise in reported cases of hepatitis among turkey farms, the USDAH considered THRVs as one of the top ten challenges affecting the turkey industry, ranking them as the ninth most serious concern [42].

A review of data from January 2019 to April 2020 indicates that cases of THRV are increasing in North America and that the disease occurs mainly in <3-week-old poults (average 15.5 days). In a similar retrospective study on turkey viral hepatitis, turkeys at younger ages were most susceptible, with an average age of 29 days [43]. However, this was based on 76 cases reported from California only. Turkey reovirus was isolated from two cases, but it was not confirmed as a causative agent of viral hepatitis. No such cases were reported from the Midwest and Eastern states until 2019, when TRV-associated viral hepatitis cases were reported in turkey poults from several states [30,31,32]. The retrospective analysis study of diagnostic submissions at MVDL from March 2010 to May 2018 reported TARV-associated lameness cases only, but no THRV [44]. This also indicates the emergence of THRVs in 2019.

The association of reovirus with turkey hepatitis is not surprising. Previously, reovirus-associated hepatitis has been reported in chicken [9,45,46,47], psittacine species [48], geese [16], Pekin ducks (*Anas platyrhynchos*) [13], magpies (*Pica pica*) [49], and American crows [50,51]. Occasional investigations into the role of reovirus in turkey hepatitis have been documented. For instance, a surveillance study conducted in Poland, which encompassed laying hens, broilers, and turkeys, identified cases of reovirus-related hepatosplenomegaly, accompanied by the presence of white necrotic foci within the tissue parenchyma [52]. Shivaprasad et al. [53] observed acute centrilobular necrosis with vacuolation of hepatocytes without inflammation in reovirus-infected turkeys. One of the birds exhibited multifocal necrosis of hepatocytes, accompanied by infiltration of lymphocytes, plasma cells, and macrophages. The present study, however, is more comprehensive; continuous submissions of these cases to our laboratory indicate that THRV can cause hepatitis in addition to arthritis and enteritis. Also, evaluating the economic significance of this newly emerging disease is necessary.

We observed hepatosplenomegaly associated with THRV in turkey poults, primarily from August to December as well as in March and May (Figure 1a). The TARV retrospective study did not find any seasonality pattern and reported that the overall probability of TARV-positive cases varied by month, with higher rates in January, April, July, and December [44]. While our study does not delve into the seasonal aspects of this disease, studying the seasonality of infection will be essential in the future to inform efforts for infection control during high-risk periods. Additional research is also needed to understand the effects of climate and management of turkey flocks on turkey reoviruses.

Interestingly, the emergence of the THRVs was consistent with the detection of the new duck reovirus (NDRV) in ducks and geese, which was also associated with hemorrhage and necrosis in the livers and spleens of 5- to 25-day-old ducklings [54]. Additionally, the NDRV infection occurred mainly at young ages; the younger the age of infected ducks, the higher the mortality and morbidity in ducklings, as seen with THRV infection in this study. Malkinson et al. [55] also reported a reovirus-associated disease in young Muscovy ducklings, which appeared as early as 10 days of age. The THRV-affected turkey flocks had a history of 2–8% mortality within a span of one week. The morbidity and mortality associated with THRVs can be highly variable [29], depending on the strain of THRV, as indicated in a pathogenicity study of two THRV isolates [33]. However, the morbidities and mortalities associated with THRVs were significantly lower than NDRV in ducks and geese, causing high morbidity (10–30%) and mortality (40–60%) [12,54].

We developed and used an ISH probe specifically designed to target avian reovirus. Positive in situ hybridization reaction demonstrated that reovirus was directly associated with characteristic hepatic lesions, thus confirming it as the etiologic agent. The ISH test developed in this study could provide an additional confirmatory tool in the field of TRV diagnosis, but after full validation. Ancillary tests such as virus isolation from these cases, virus detection by rRT-PCR and electron microscopy, and our previous study on pathogenesis [33] further confirm the reovirus etiology of turkey hepatitis. All these ancillary diagnostic observations agree with the data reported in the published literature on duck, chicken, and turkey reovirus concerning molecular detection, virus isolation in embryonated eggs and cell culture, ISH, and EM [2,56]. In this study, we also conducted nucleotide and amino acid sequence analysis of the complete σC protein of the THRVs, using representative sequences of chicken and turkey reoviruses based on the common σC classification of ARVs, as conducted by Kant et al. [7]. Recently, Seller et al. [8] have refined avian reovirus classification into the seven main genetic clusters (GCs), with the majority of the TRVs belonging to GC 2. THRVs isolated in this study, like other TRVs, were positioned in the same GC 2, with high divergence from GC 1, which contains classical avian reoviruses and vaccinal strains (S1133 and 1733) [18,37,57,58]. Notably, THRVs were more similar to TARVs, such as the reference TARV-O’Neil and other TARVs, with a maximum identity of 98–99%. THRVs were divided into four groups based on distinct monophyletic groups, intra- and inter-group average identity percentage, and characteristic aa substitutions for each group. Specifically, these amino acid substitutions were variable among the groups, ranging from nine to seven in the more divergent THRV groups (groups I and IV) versus only two unique substitutions in groups II and III. These consistent aa substitutions were exclusive to the THRV groups (absent in all TARVs and TERVs); therefore, they could act as genetic markers distinguishing THRVs from TARVs and TERVs. These unique aa substitutions may be related to the tissue tropism and virulence of THRVs.

The molecular characterization of all THRVs isolated in this study indicates their close relation to TARVs, with maximum identity to the TARV O’Neil strain, suggesting that THRVs are variants of TARV with increased pathogenicity. This is also supported by the pathogenicity study of Kumar et al. [33], who stated that THRVs can cause tenosynovitis in addition to hepatitis. Similarly, chicken arthritis strains like 176, UM 1–203 were reported to cause necrotizing hepatitis and splenitis with high mortality after 48 to 72 h post-infection [46,59]. The σC exhibited the highest rate of sequence divergence and rapid evolution and possessed the type- and broad-specific neutralizing epitopes; therefore, the σC gene could be used as a genetic marker for rapid differentiation between novel THRV and TARV. In a comparative pathogenesis study, Kumar et al. [33] reported that not all THRVs are highly pathogenic, which may be correlated with the differences in amino acid changes among different groups of THRVs. Hence, there is a need to correlate genetic mutations in these groups with pathogenicity for a better understanding of which groups are highly pathogenic.

## 5. Conclusions

In conclusion, the results of the present study suggest that mortality in young poults was due to multifocal necrotizing hepatitis caused by reovirus infection. This appears to be an emerging disease caused by a previously recognized pathogen. To the best of our knowledge, this study represents the first phylogenetic analysis of the novel THRVs that emerged recently in commercial turkey farms. It revealed that based on σC, the THRVs isolated from NA commercial turkey farms can be classified into four distinct groups, exhibiting characteristic amino acid changes that distinguish them from CRVs, TARVs, and TERVs. THRVs are more related to most TARVs, with some TARVs displaying a significant degree of identity to certain THRV groups. Further comparative pathogenicity studies are crucial, particularly between representatives from different groups and closely related TARVs, to confirm the genetic marker behind the liver tropism of THRVs and understand evolutionary patterns. Phylogenetic analysis based on whole-genome sequencing and more divergent segments, such as M2 and L3, is also essential for a broader understanding and robust genetic characterization of these emerging reovirus hepatitis variants.

## Figures and Tables

**Table 1:** The accession numbers and geographical distributions of the THRVs isolated in this study and used in phylogenetic analysis.

Sequence Name	GenBank Accession Numbers	Country	State	Age (Days)
MVDL-THRV-1/USA/AR/2019	OR525778	United States	Arkansas	14
MVDL-THRV-2/USA/AR/2019	OR525777	United States	Arkansas	22
MVDL-THRV-3/USA/AR/2019	OR525788	United States	Arkansas	-
MVDL-THRV-4/USA/AR/2019	OR525787	United States	Arkansas	21
MVDL-THRV-5/Canada/2019	OR525775	Canada	-	-
MVDL-THRV-6/USA/MN/2019	OR525782	United States	Minnesota	14
MVDL-THRV-7/USA/MN/2019	OR525786	United States	Minnesota	-
MVDL-THRV-8/USA/MN/2019	OR525789	United States	Minnesota	14
MVDL-THRV-9/USA/MN/2019	OR525783	United States	Minnesota	8
MVDL-THRV-10/USA/MO/2020	OR525790	United States	Missouri	14
MVDL-THRV-11/USA/MO/2020	OR525784	United States	Missouri	18
MVDL-THRV-12/USA/AR/2020	OR525785	United States	Arkansas	12
MVDL-THRV-13/USA/MO/2020	OR525776	United States	Missouri	-
MVDL-THRV-14/USA/KY/2020	OR525794	United States	Kentucky	13
MVDL-THRV-15/USA/VA/2020	OR525795	United States	Virginia	10
MVDL-THRV-16/USA/VA/2020	OR525796	United States	Virginia	10
MVDL-THRV-17/USA/IN/2020	OR525793	United States	Indiana	15
MVDL-THRV-18/USA/MN/2021	OR525791	United States	Minnesota	10
MVDL-THRV-19/USA/IN/2021	OR525797	United States	Indiana	11
MVDL-THRV-20/Canada/2021	OR525792	Canada	-	10
MVDL-THRV-21/Canada/2021	OR525774	Canada	-	4
MVDL-THRV-22/Canada/2021	OR525773	Canada	-	13
MVDL-THRV-23/USA/IA/2021	OR525780	United States	Iowa	-
MVDL-THRV-24/USA/IA/2021	OR525781	United States	Iowa	23
MVDL-THRV-25/USA/VA/2021	OR525779	United States	Virginia	-

**Table 2:** Submission of cases from different geographical locations in the US and Canada.

Country	State (Codes)	Submitter	No. of Submissions	No. (%) Positive for Reovirus
US	Arkansas (AR)	Client 1	8	8 (100)
	California (CA)	Client 2	1	0
	Indiana (IN)	Client 3	3	2 (67)
	Iowa (IA)	Client 4	9	0
		Client 5	2	2 (100)
	Michigan (MI)	Client 6	1	0
	Minnesota (MN)	Client 7	1	1 (100)
		Client 8	1	1 (100)
		Client 9	1	0
		Client 10	8	3 (37.5)
		Client 11	54	36 (67)
		Client 12	15	12 (80)
		Client 13	3	0
		Client 14	3	1 (33)
		Client 15	36	6 (17)
	Missouri (MO)	Client 16	7	6 (86)
		Client 17	1	0
	Nebraska (NE)	Client 18	1	0
	North Carolina (NC)	Client 19	3	1 (33)
	Wisconsin (WI)	Client 20	18	9 (50)
Canada	NA	Client 2	8	0
	NA	Client 9	1	0
Total			185	88 (47.5)

**Table 3:** Distribution of total samples and positive cases by age group.

Age (Days)	No. of Submissions	No. (%) Positive for Reovirus
0–6	69	7
7 to 14	60	42
15 to 21	35	27
22 to 28	9	7
29 to 35	7	2
36 to 42	1	1
43 to 49	2	1
>50	2	1
Total	185	88

**Table 4:** The identity percentage between the four groups of turkey hepatitis reovirus (THRV) isolates and turkey arthritis reoviruses (TARVs), turkey enteric reoviruses (TERVs), and chicken reoviruses based on the Sigma C (σC) gene.

			Group 1		Group 2		Group 3		Group 4	
			NA	AA	NA	AA	NA	AA	NA	AA
Genocluster 1	Subcluster I	THRV Group I	99.6	99.5	96.5	95.3	96.4	95.9	96.3	96.0
		THRV Group II	96.5	95.3	99.0	99.0	97.0	97.4	96.7	94.0
		THRV Group III	96.4	95.9	97.7	97.4	99.6	99.6	97.1	96.0
		THRV Group IV	96.3	95.2	96.7	96.2	97.1	96.7	99.4	100
		TARV-O’ Neil-like sequences	97.0	97.4	98.0	98.1	98.7	98.2	98.3	97.5
		Reo/Turkey/PA/00659/14	97.2	95.6	97.8	97.3	98.2	98.0	98.3	98.1
		Reo/Turkey/PA/01769/14 Reo/Turkey/PA/27011/13	97.0	96.0	98.3	98.4	97.8	98.2	96.8	97.4
		Reo/PA/Turkey/27399/12 Reo/PA/Broiler/27541/12	96.9	95.9	98.0	97.6	98.4	98.4	97.7	97.0
		Tk_SD_121541_Td_2016	98.7	99.0	96.3	95.3	96.4	96.1	96.2	95.1
		Tk_SD_121539_Td_2016 Tk_SD_121540_Td_2016 Tk_SD_121542_Td_2016 Tk_SD_121544_Td_2016	94.8	93.6	95.1	94.8	95.2	95.4	95.5	94.7
		ARV/TK/HUN/19831M09 ARV/TK/HUN/D1246/09 ARV/TK/HUN188/16	94.2	93.8	95.4	95.1	95.4	95.8	95.6	95.0
		TERV-MN1	95.2	93.9	95.1	95.8	96.1	96.4	96.3	95.5
		TRV/NC/SEP-R61/03	93.2	92.6	93.8	94.4	93.9	95.0	94.4	94.8
	Subcluster II		90.0	91.0	91.6	91.6	91.6	91.9	92.1	91.6
	Subcluster III		86.7	85.7	87.3	87.3	87.0	87.3	87.1	86.1
Genocluster 2			59.4	53.7	59.6	54.9	59.6	54.2	59.6	54.2

## Data Availability

The original contributions presented in this study are included in the article. Further inquiries can be directed to the corresponding author.

## Abbreviations

The following abbreviations are used in this manuscript:

ARVs	Avian reoviruses
CPE	Cytopathic effects
EM	Electron microscopy
FFPE	Formalin fixed paraffin embedded
H&E	Hematoxylin and Eosin
HBSS	Hanks’ balanced salt solution
ISH	In situ hybridization
MVDL	Minnesota Veterinary Diagnostic Laboratory
rRT-PCR	Real-time reverse transcription-PCR
TARV	Turkey arthritis reovirus
TERV	Turkey enteric reovirus
THRV	Turkey hepatitis reovirus

## References

[1] Spandidos D.A., Graham A.F. (1976). Physical and chemical characterization of an avian reovirus. J. Virol..

[2] Jones R.C., Saif Y.M. (2009). Viral arthritis. Diseases of Poultry.

[3] Varela R., Benavente J. (1994). Protein coding assignment of avian reovirus strain S11343. J. Virol..

[4] Schnitzer T.J., Ramos T., Gouvea V. (1982). Avian reo virus polypeptides: Analysis of intracellular virus-specified products virions, top component, and cores. J. Virol..

[5] Martínez-Costas J., Grande A., Varela R., García-Martínez C., Benavente J. (1997). Protein architecture of avian reovirus S1133 and identification of the cell attachment protein. J. Virol..

[6] Wickramasinghe R., Meanger J., Enriquez C.E., Wilcox G.E. (1993). Avian reovirus proteins associated with neutralization of virus infectivity. Virology.

[7] Kant A., Balk F., Born L., van Roozelaar D., Heijmans J., Gielkens A., ter Huurne A. (2003). Classification of Dutch and German avian reoviruses by sequencing the sigma C protein. Vet. Res..

[8] Sellers H.S. (2022). Avian reoviruses from clinical cases of tenosynovitis: An overview of diagnostic approaches and 10-year review of isolations and genetic characterization. Avian Dis..

[9] Rosenberger J.K., Sterner F.J., Botts S., Lee K.P., Margolin A. (1989). In vitro and in vivo characterization of avian reoviruses. I. Pathogenicity and antigenic relatedness of several avian reovirus isolates. Avian Dis..

[10] Tang Y., Lu H. (2015). Genomic characterization of a broiler reovirus field strain detected in Pennsylvania. Infect. Genet. Evol..

[11] Chen Z., Zhu Y., Li C., Liu G. (2012). Outbreak-associated novel duck Reovirus, China, 2011. Emerg. Infect. Dis..

[12] Wang H., Gao B., Chen H., Diao Y., Tang Y. (2019). Isolation and characterization of a variant duck orthoreovirus causing spleen necrosis in Peking ducks, China. Transbound. Emerg. Dis..

[13] Liu Q., Zhang G., Huang Y., Ren G., Chen L., Gao J., Zhang D., Han B., Su W., Zhao J. (2011). Isolation and characterization of a reovirus causing spleen necrosis in Pekin ducklings. Vet. Microbiol..

[14] Niu X., Tian J., Yang J., Jiang X., Wang H., Tang Y., Diao Y. (2018). Complete genome sequence of a novel avian orthoreovirus isolated from gosling, China. Arch. Virol..

[15] Yang J., Tian J., Chen L., Tang Y., Diao Y. (2018). Isolation and genomic characterization of a novel chicken-origin orthoreovirus causing goslings hepatitis. Vet. Microbiol..

[16] Palya V., Glávits R., Dobos-Kovács M., Ivanics É., Nagy E., Bányai K., Szücs G., Dá Á., Benkö M. (2003). Reovirus identified as cause of disease in young geese. Avian Pathol..

[17] Tang Y., Lu H., Sebastian A., Yeh Y.T., Praul C.A., Albert I.U., Zheng S.Y. (2015). Genomic characterization of a turkey reovirus field strain by next-generation sequencing. Infect. Genet. Evol..

[18] Lu H., Tang Y., Dunn P.A., Wallner-Pendleton E.A., Lin L., Knoll E.A. (2015). Isolation and molecular characterization of newly emerging avian reovirus variants and novel strains in Pennsylvania, USA, 2011–2014. Sci. Rep..

[19] Mor S.K., Sharafeldin T.A., Abin M., Kromm M., Porter R.E., Goyal S.M., Patnayak D.P. (2013). The occurrence of enteric viruses in light turkey syndrome. Avian Pathol..

[20] Guy J.S., Levy M.G., Ley D.H., Barnes H.J., Gerig T.M. (1987). Experimental reproduction of enteritis in bobwhite quail (*Colinus virginianus*) with *Cryptosporidium* and reovirus. Avian Dis..

[21] Hollmen T., Docherty D.E., Thomas N.C., Hunter D.B., Atkinson C.T. (2007). Orthoreovirus. Infectious Diseases of Wild Birds.

[22] Styś-Fijoł N., Kozdruń W., Czekaj H. (2017). Detection of avian reoviruses in wild birds in Poland. J. Vet. Res..

[23] Pantin-Jackwood M.J., Day J.M., Jackwood M.W., Spackman E. (2008). Enteric viruses detected by molecular methods in commercial chicken and turkey flocks in the United States between 2005 and 2006. Avian Dis..

[24] Jindal N., Patnayak D.P., Chander Y., Ziegler A.F., Goyal S.M. (2010). Detection and molecular characterization of enteric viruses from poult enteritis syndrome in turkeys. Poult. Sci..

[25] Al Afaleq A., Jones R.C. (1989). Pathogenicity of three turkey and three chicken reoviruses for poults and chicks with particular reference to arthritis/tenosynovitis. Avian Pathol..

[26] Levisohn S., Gur-Lavie A., Weisman J. (1980). Infectious synovitis in turkeys: Isolation of tenosynovitis virus-like agent. Avian Pathol..

[27] Page R.K., Fletcher O.J., Rowland G.N., Gaudry D., Villegas P. (1982). Malabsorption syndrome in broiler chickens. Avian Dis..

[28] Mor S.K., Sharafeldin T.A., Porter R.E., Ziegler A., Patnayak D.P., Goyal S.M. (2013). Isolation and characterization of a turkey arthritis reovirus. Avian Dis..

[29] USAHA-2021. https://www.eatturkey.org/wp-content/uploads/2020/01/Economic-Impact-of-TARV_NTF_122019.pdf.

[30] Lighty M. Viral Hepatitis in Turkey Poults. Proceedings of the 63rd American Association of Avian Pathologists (AAAP) Virtual Annual Meeting.

[31] Porter R. Characterization of Reoviral Hepatitis and Splenitis in Commercial Turkey Poults. Proceedings of the 63rd American Association of Avian Pathologists (AAAP) Virtual Annual Meeting.

[32] Smith A. Turkey Reovirus Hepatitis: An Emerging Disease from a Known Etiological Agent. Proceedings of the 63rd American Association of Avian Pathologists (AAAP) Virtual Annual Meeting.

[33] Kumar R., Sharafeldin T.A., Sobhy N.M., Goyal S.M., Porter R.E., Mor S.K. (2022). Comparative pathogenesis of turkey reoviruses. Avian Pathol..

[34] Wang F., Flanagan J., Su N., Wang L.C., Bui S., Nielson A., Wu X., Vo H.T., Ma X.J., Luo Y. (2012). RNAscope: A novel in situ RNA analysis platform for formalin-fixed, paraffin-embedded tissues. J. Mol. Diagn..

[35] Frey M.C., Hanson R.P., Anderson D.P. (1968). A medium for the isolation of avian *Mycoplasma*. Am. J. Vet. Res..

[36] Armién A.G., Wolf T.M., Mor S.K., Ng T.F.F., Bracht A.J., Goyal S.M., Rasmussen J.M. (2020). Molecular and Biological Characterization of a Cervidpoxvirus Isolated From Moose with Necrotizing Dermatitis. Vet. Pathol..

[37] Gál B., Varga-Kugler R., Ihász K., Kaszab E., Domán M., Farkas S., Bányai K. (2023). Marked Genotype Diversity among Reoviruses Isolated from Chicken in Selected East-Central European Countries. Animals.

[38] Sievers F., Higgins D.G. (2018). Clustal Omega for making accurate alignments of many protein sequences. Protein Sci..

[39] Kearse M., Moir R., Wilson A., Stones-Havas S., Cheung M., Sturrock S., Buxton S., Cooper A., Markowitz S., Duran C. (2012). Geneious Basic: An integrated and extendable desktop software platform for the organization and analysis of sequence data. Bioinformatics..

[40] Tamura K., Stecher G., Kumar S. (2021). MEGA11: Molecular Evolutionary Genetics Analysis Version 11. Mol Biol Evol..

[41] Mor S.K., Kumar R., Sobhy N.M., Singh A., Kakrudi N., Marusak R.A., Goyal S.M., Porter R.E. (2020). Enteric Viruses Associated with Mid-growth Turkey Enteritis. Avian Dis..

[42] Clark S.R., Chiaia L. Current Health and Industry Issues Facing the US Turkey Industry (HVP.PO.100324.1). Proceedings of the 128th Annual Mtg of the USAHA, Committee on Poultry and Other Avian Species.

[43] Hauck R., Chin R.P., Sentíes-Cué G., Charlton B., Shivaprasad H.L. (2014). Retrospective study of Turkey Viral Hepatitis in California turkey flocks, 2000–2012. Avian Dis..

[44] Barrera M., Kumar P., Porter R.E., Goyal S.M., Mor S.K. (2019). Retrospective Analysis of Turkey Arthritis Reovirus Diagnostic Submissions in Minnesota. Avian Dis..

[45] Chénier S., Boulianne M., Gagnon C.A. (2014). Postvaccinal reovirus infection with high mortality in breeder chicks. Avian Dis..

[46] Tang K.N., Fletcher O.J., Villegas P. (1987). The effect on newborn chicks of oral inoculation of reovirus isolated from chickens with tenosynovitis. Avian Dis..

[47] Tang K.N., Fletcher O.J., Villegas P. (1987). Comparative study of the pathogenicity of avian reoviruses. Avian Dis..

[48] van den Brand J.M., Manvell R., Paul G., Kik M.J., Dorrestein G.M. (2007). Reovirus infections associated with high mortality in psittaciformes in The Netherlands. Avian Pathol..

[49] Lawson B., Dastjerdi A., Shah S., Everest D., Núñez A., Pocknell A., Hicks D., Horton D.L., Cunningham A.A., Irvine R.M. (2015). Mortality associated with avian reovirus infection in a free-living magpie (*Pica pica*) in Great Britain. BMC Vet. Res..

[50] Forzán M.J., Renshaw R.W., Bunting E.M., Buckles E., Okoniewski J., Hynes K., Melendez R., Ableman A., Laverack M., Fadden M. (2019). A novel orthoreovirus associated with epizootic necrotizing enteritis and splenic necrosis in American crows (*Corvus brachyrhynchos*). J. Wildl. Dis..

[51] Meteyer C., Docherty D., Ip H., Ramsay N., Saito E., Oaks L. Reovirus-associated necrotizing enteritis in American crows. Proceedings of the Program and Abstracts of the 58th Annual International Conference of the Wildlife Disease Association.

[52] Czekaj H., Kozdruń W., Styś-Fijoł N., Niczyporuk J.S., Piekarska K. (2018). Occurrence of reovirus (ARV) infections in poultry flocks in Poland in 2010–2017. J. Vet. Res..

[53] Shivaprasad H.L., Franca M., Woolcock P.R., Nordhausen R., Day J.M., Pantin-Jackwood M. (2009). Myocarditis associated with reovirus in turkey poults. Avian Dis..

[54] Yu K., Ti J., Lu X., Pan L., Liu L., Gao Y., Guo X., Hu F., Liu C., Ma X. (2021). Novel duck reovirus exhibits pathogenicity to specific pathogen-free chickens by the subcutaneous route. Sci. Rep..

[55] Malkinson M., Perk K., Weisman Y. (1981). Reovirus infection of young Muscovy ducks (*Cairina moschata*). Avian Pathol..

[56] Markis M., Rosenberger J.K., Williams S.M. (2016). Viral arthritis/tenosynovitis and other reovirus infections. A Laboratory Manual for the Isolation, Identification, and Characterization of Avian Pathogens.

[57] Day J.M., Pantin-Jackwood M.J., Spackman E. (2007). Sequence and phylogenetic analysis of the S1 genome segment of turkey-origin reoviruses. Virus Genes.

[58] Mor S.K., Verma H., Sharafeldin T.A., Porter R.E., Jindal N., Ziegler A., Goyal S.M. (2014). Characterization of S class gene segments of a newly isolated turkey arthritis reovirus. Virology.

[59] Mandelli G., Rampin T., Finazzi M. (1978). Experimental reovirus hepatitis in newborn chicks. Vet. Pathol..

